# Characteristics of Alloys

**Published:** 1889-03

**Authors:** J. P. Watson

**Affiliations:** U. of M.


### Characteristics of Alloys.

BY J. P. WATSON, U. OF M.

To alloy is to change the purity of a metal by admixture with another.

Alloys are a class of compounds in which may be observed the general characteristics, to a greater or less degree, of the metals forming the compound. They are not only mixtures of metals possessing certain distinct properties, but are true chemical compounds, and are formed to fill a demand, the pure metals are unable to meet.

As the number and character of metallic compounds are innumerable, any classification of them would necessarily be imperfect, still, for all practical purposes, the classification suggested by Matthiesen is perhaps the most generally accepted.

He regards an alloy as either a soldified solution of one metal in another, a chemical combination, a mechanical mixture, or a solution or mixture of two or all of the above.

That such conclusions are warranted is evident in the study of the behavior of various metals toward each other when being formed into an alloy.

In the phenomena, which accompanies the union of certain metals, we observe the action which characterizes chemical affinity, such as heat, etc., resulting in compositions having definite characteristics, and differing from those of the constituents. The affinity of an alloy for Oxygen is greater than that of its separate metals. Although there is no distinct boundary between the laws governing the forming of solutions, and those of chemical combinations. Yet there are marked differences in the results produced. We find a chemical compound possesses properties entirely different from the elements of which it is composed. For illustration, Hydrogen an inflammable gas, Oxygen a gaseous body, and energetic supporter of combustion, when united produce $H_2O$. which is neither inflammable or combustible.

A characteristic feature of all chemical compounds is that they unite in exact proportions by weight, indifferent as to the quantities that may be brought together. On the other hand. in solutions, where some of the properties of the constituents are to a degree lost, or modified, they always possess, to a greater or less extent, the properties of the substances which went to form the solution.

This is true in all solutions, while it is directly the reverse in chemical compounds.

Examples of metals combined in definite proportions are found in nature in the alloy of Au. and Ag.

All metals do not unite indifferently with each other, but are controlled by affinities, for example. Ag. combines readily with Au., Cu. and Sn. but not with Fe.

The peculiar affinities of certain metals for each other, is well illustrated in the manufacture of alloys of Cu., Sn. and Sb. If 10 parts of Sb. be added to 90 parts of Cu. and 10 of Sn., an alloy will be produced, differing markedly from one made by adding 90 parts of Cu. to 10 parts of Sn. and 10 of Sb. In each the metals are the same and united in the same proportions, yet in the first Sb. is added to a compound of Cu. and Sn. A Cu. and Sn. compound holding Sb. in solution, while in the other case Cu. was added to a Sb., Sn. compound, a Sn., Sb. compound holding Cu. in solution, these furnishing alloys of widely differing physical properties.

It must not be assumed, however, that all alloys employed are the result of definite combination, dissolved in excess of one of the metals. Many combinations are capable of co-existing in the same alloy. This can be demonstrated in the alloy of Sn., Sb. and Bi. which melts below the boiling point of $H_2O$. heated to 25°c., and then permitted to cool, will be observed by the use of the thermometer, the fall of the temperature is twice distinctly arrested. The cause of this phenomena has been assumed to be the production of a less fusible alloy, which in solidifying, evolves heat, retarding for a time the gradual cooling of the mass.

Alloys are generally harder and more fusible than the metals of which they are formed. Many metals, owing to their softness or high fusing points, are unfit for use in the arts, their properties are so modified to suit various requirements by combinations with other metals.

It is often desirable to unite two, or more, pieces of metal, or metals. This is accomplished by an alloy, called solder, formed of the metal on which it is to be used, with the addition of another metal that will reduce its fusing point without affecting its color.

The changes in the physical properties of metals in alloying, can not be anticipated before experimenting; the slightest variations producing marked changes in the properties of the product.

The specific gravity of alloys varies greatly and may be either, greater or less than the means of the specific gravities of their component metals.

According to Prof. Essig, the chief characteristics of alloys are enumerated as follows:

Density, Color, Malleability, Ductility, Tenacity, Fusibility, Composition, Decomposition, Influence of Constituent Metals, Liqudation, Temper, and Preparation.

The DENSITY of an alloy, theoretically would be supposed to be the mean of its constituents. Such is not the case, it is sometimes equal to, or greater, or less than the theoretical mean.

COLOR is always modified, still it is such as would be expected from the metals entering into the mixture. 3 parts of Ag. to 7 of Au. is an exception, giving a green alloy and nickel added to brass gives an alloy of silver whiteness.

MALLEABILITY, DUCTILITY AND TENACITY, are properties depending upon molecular cohesion malleability depends upon the capacity of being drawn out by beating. Ductility, that of being drawn into a wire. Tenacity, that of firmly holding together. Malleability and Ductility are not only greatly modified by alloying, but in some instances are completely destroyed, even when in combination with two very ductile metals, as Au. and Pb. $\frac{1}{1900}$ part of Sb. to Au. makes the Au. quite brittle, on the other hand, tenacity is generally greatly increased by alloying. For example, Cu. alloyed with 10 per cent. of Sn. increases its tensile strength to 10,000 pounds per square inch, and alloyed with Cu. has its tenacity increased nearly 100 per cent.

FUSIBILITY.

The relation of the fusing point of alloys to those of their constituent metals is variable and cannot be predicted with certainty, yet it is always less than that of the least fusible metal contained in the alloy. An alloy of 5 parts of Bi., 3 parts of Pb., and 2 parts of Sn., melts at 90°c., less than the boiling point of $H_2O$, while Sn. alone fuses at 227 .8°c., and Pb. at 325°c.

Pure Fe.s fusibility requires an intense heat, a little less than 3,000°, yet, when combined with a proper amount of As. and P., may be melted in a cast iron pot without adhering to it. This phenomena as explained by Matthieson, is as follows: Matter in a solid state exhibits excess of attraction over repulsion, while in the liquid state these forces are balanced, and in the gaseous state repulsion predominates over attraction, and similar particles of matter attract each other more powerfully than dissimilar particles do. The attraction subsisting between the particles of a mixture will be sooner overcome by repulsion than will the attraction in the case of a homogeneous body; hence mixtures should fuse more readily than their constituents.

Decomposition takes place in the application of heat to an alloy containing a volatile metal, and to expel the last trace of the volatile element, requires a temperature greatly higher than the metals normal temperature of melting.

INFLUENCES OF CONSTITUENT METALS.

While marked differences may exist between the properties of alloys, and the metals forming them, still there are certain metals, that when placed in combination, give to it definite qualities, for instance: Hg., Cd. and Bi., increase fusibility. Sn. hardens and gives tenacity. As. and Sb. produce brittleness. When As., or arsenic compounds unite with alloys of Cu. and Sn., known as bronze or gun-metal, it gives to them several remarkable and desirable qualities, namely, homogeneity, hardness, elasticity, toughness, great density, and greatly increased tensile strength.

All oxidizable metals, when fused, have the power of dissolving the oxides formed by contact of air with its melted surface, its result being to produce an alloy greatly deficient in strength and toughness. The injurious influence of dissolved oxides in Cu. and Sn. alloys is amply illustrated in Levy & Kunkel's experiments. This alloy after contact with the air for some time, in the molten state, was cast, and found to have a tensile strength of 22,982 lbs. per square inch, while the same alloy completely deoxidized by a minute quality of P. had a tensile strength of 33,916 lbs. per square inch., a gain of nearly 11,000 lbs. of cohesive power over the alloy containing dissolved oxide. This same detrimental effect is seen in the case of melting tin for dies for dental work. After repeated meltings with free access to air, it becomes thick and will not fill out the mould sharply; yet a small quantity of anything rich in C. thrown upon its surface, restores its former working qualities.

Liquation is the separation by regulated heat, of an easily fusible metal from one less fusible. When the constituent of an alloy is heated to near its melting point, they frequently unite to form new compounds, and if the fluid portion is poured off a solid alloy remains less fusible than the original. Cu. and Ag. are separated in this manner.

Temper, modified hardness and elasticity of metals is often desired; this can be produced by an admixture with other metals, then cooling rapidly, or slowly, as experience suggests. An alloy of 94 parts of Cu. to 6 of Sn. forms an extremely brittle compound if cooled slowly, but when chilled suddenly is quite malleable.

In the preparation of alloys, when a noble metal is to be united with one or more of the baser metals, the noble metal should be fused first, then add the baser metal, and cover the surface with any substance that will prevent oxidation. The same method should be pursued in reducing the fusible point of Au. and Ag. for solder.

The action of acids is usually more energetic on alloys than on the individual metal, forming the compound. Yet, sometimes quite the opposite is observed. Ag. alloyed with a large amount of Au. is protected from the action of $H.N.O._3$ in the one case, while in the other, Pt. insoluble in $H.N.O._3$ is readily dissolved when sufficiently alloyed with Ag.

